# Motherhood, the “blank slate,” and the language of surrogacy

**DOI:** 10.3389/fgwh.2026.1761415

**Published:** 2026-05-19

**Authors:** Karleen D. Gribble

**Affiliations:** School of Nursing and Midwifery, Western Sydney University, Parramatta, NSW, Australia

**Keywords:** adoption, child rights, donor conception, language, motherhood, surrogacy

## Abstract

Women who gestate and give birth to infants or who provide oocytes in surrogacy arrangements are most often described as “surrogates,” “gestational carriers,” or “donors,” rather than mothers. This terminology is used not only by the women themselves but also by those wishing to become parents via surrogacy and by those who profit from surrogacy. Caregiving parents predominantly discourage their children from using language that ascribes parenthood to those who gestated and gave birth to them or provided the gametes for their conception. In rationalising this linguistic choice, the importance of gestation, birth, and genetics is downplayed. This perspective paper discusses the significance of genetic and gestational motherhood for children born via surrogacy. In doing this, it explores the foetal and neonatal experiences of gestational motherhood. Insights from adoption and donor conception are also presented, highlighting the limitations and harms of treating infants as “blank slates.” In addition, the paper reviews research on the language used by individuals born via surrogacy and donor conception to describe the women who gestated and gave birth to them or those who provided the gametes for their conception. It is concluded that it is beneficial for children for the language of surrogacy to recognise the multiplicity of their mothers.

## 1 Introduction

Surrogacy is a form of third-party reproduction in which women intentionally gestate and give birth to infants for others to raise. It often involves women providing oocytes for the creation of embryos. While such women are biological mothers, via genetics and/or gestation, to the resulting children, this relationship is often obscured in the language used to describe them (1). Instead of being described as “mothers,” they are most commonly referred to as “surrogates” or “gestational carriers” (when they gestate and give birth) or “donors” (where they provide oocytes). Drawing on research from surrogacy, donor conception, and adoption, this paper discusses the significance of the language used to refer to women who gestate and give birth to infants or provide oocytes in surrogacy arrangements and places explicit focus on the experiences, needs, and viewpoints of children born via surrogacy.

## 2 Rejection of motherhood helps facilitate surrogacy involvement

Women who gestate and give birth to infants in surrogacy arrangements are amongst those who deny that they are mothers (2, 3). In addition to describing themselves as “surrogates” or “gestational carriers,” they may also refer to themselves as “caretakers,” “babysitters,” “custodians,” “hosts,” or even “ovens,” and emphasise that they are “not the actual mother” (2, 3). In cases where the oocyte is provided by another woman, those undertaking surrogacy may reject a mother–child relationship on the basis of a lack of genetic relatedness (2, 4). However, even when the child is conceived using the woman's own oocyte, she may still maintain that she is not a mother to the child (4, 5).

Similarly, women who provide oocytes for donor conception, including in surrogacy arrangements, commonly reject that they are mothers. They may conceptualise their provision as analogous to donating blood or describe oocytes as no different from skin cells, thereby distancing themselves from any resulting child (6, 7). In addition, or alternatively, they may construct motherhood as arising from gestation or caregiving rather than genetics (6, 7) or they may downplay the significance of genetic relatedness (8).

Those wishing to become parents via surrogacy also argue that motherhood should be defined in terms of caregiving or genetics (the latter only if a woman's own oocyte is used to conceive the child) (9–11). They therefore tell themselves “I **am** the mother…[she is] my carrier” [(12), p. 82, emphasis in original]. Women who become mothers via surrogacy have expressed that referring to the gestating and birthing woman as a mother diminishes their own maternal status (11). However, the motherhood of those who gestate and give birth may be rejected even when the contracting parents are single men or male couples (13, 14). As one gay father via surrogacy stated, “the term mother means ‘care’ and more than carrying a child and giving birth…we don't view the surrogate as a mother to our child” [(10), p. 13].

Those who profit from surrogacy, promote the non-motherhood of women who gestate and birth infants in order to facilitate their business. Surrogacy agency websites inform individuals considering surrogacy, whether for family formation or for gestating and giving birth to a child for others, that the pregnant woman is not the mother (15–17). This conceptualisation is maintained post-conception, as one account notes, “[she] is not the mother, she is only the ‘carrier’ of the baby, and she is constantly reminded of this by the clinic” [(9), p. 319]. Some have even sought to police language within medical discourse (18). While, in many jurisdictions, a woman who gestates an infant in a surrogacy arrangement is recognised as the legal mother at birth (19), her motherhood may be contested if she seeks to retain care of the child (20).

The original nomenclature for women who give birth to infants for others was “surrogate mother” (1). While this term includes the word “mother,” the prefix “surrogate” was intended to convey that the woman was not truly a mother to the child, but merely a stand-in (1, 21). Nonetheless, the use of “surrogate mother” has declined over time, and an assessment of websites of 10 surrogacy agencies and clinics across six countries found that the term “surrogate” alone was used 50% more frequently than “surrogate mother” (22). For example, one agency described that using “gestational surrogate” or “gestational carrier” rather than “surrogate mother” provides “clarity to everyone involved: the intended parents, the woman carrying their baby, and even the baby as he or she grows up and learns about how they came into the world..[that she] is not the mother of the baby…in any way” (16).

Women who would consider themselves to be mothers to children resulting from their oocyte provision are unlikely to be willing to donate or sell oocytes for another woman to become pregnant (23). Thus, fertility clinics have been described as working with women who are potential providers “to make it clear that the relationship between the donor and the potential child is not a mother/child relationship” [(24), p. 242].

## 3 How parents speak to children

The limited research on origin disclosure to children born via surrogacy suggests that parents are very likely to be honest with children about them having been gestated and birthed by another woman (25, 26). However, this woman may not be presented as a mother but instead described as “an aunt,” a “good fairy,” “a friend,” “an oven,” or “not family” (11, 25, 27). As one researcher described, caregiving mothers engage in “re-establishing reality” by explaining to children that “they are the mother, and surrogates helped them become one” [(11), p. 155].

Informing children born via surrogacy about donor oocyte conception appears to be less common than disclosing information about gestation and birth (25, 26). The involvement of another woman as the oocyte provider can be believed as a threat to the caregiving mother–child relationship, thereby discouraging disclosure (28). Non-disclosing caregiving parents may also “come to view the genetic element as almost irrelevant, a biological detail” [(25), p. 493]. Thus, many children born via donor conception and surrogacy may remain unaware of their genetic maternal origins.

Research on how parents who disclose genetic origins in surrogacy arrangements describe the woman who provided the oocyte to their children remains limited. Research on donor conception generally indicates that parents usually use language that creates distance between gamete providers and the child and avoid terms such as “mother” and “father” (29–31). Instead, genetic parents may be described as “donors” or “biological contributors,” as a “kind woman”/“man,” “a friendly mister,” as individuals who gave or lent something, such as “seeds,” or they may be depersonalised altogether, with sperm or oocytes presented as coming from a clinic rather than from an individual (27, 29–31). In some cases, fertility clinics actively encourage parents to avoid language that ascribes maternity or paternity to gametes providers, as part of a process described as “de-kinning” (27), for example, “the donor should be presented as a donor who gave the gift of life, but who is not a mother” [(32), p.483].

## 4 Foetal and infant experience of gestational motherhood in surrogacy

In surrogacy arrangements, women who gestate foetuses conceived using the oocyte of another woman are presented as little more than incubators and are insignificant beyond providing the physical needs of the foetus (33). However, this characterisation disregards the relationship and minor genetic connections that develop between the foetus and the gestating woman during pregnancy. Foetuses become familiar with the voice of the woman gestating them and show a preference for her voice over those of other women and their father, both during pregnancy and after birth (34, 35). At birth, infants can recognise the smell of the woman who gestated them and preferentially orientate towards samples of her amniotic fluid compared with that of another woman (36, 37). Newborn infants also prefer the scent of breast secretions of their mother over those of other women (38). Newborns who are separated from their mother cry less when exposed to the scent of her amniotic fluid and cry more when exposed to the scent of her breast secretions (39). Together, these findings indicate that the woman who gestated and gave birth to the infant is known to them at birth, even if she did not provide the oocyte from which they were conceived.

In addition, minor genetic relatedness develops between women and foetuses during gestation. A bidirectional flow of cells occurs between the foetus and the pregnant woman, with these cells (including their DNA) integrating into each other’s bodies in a process known as faetal–maternal and maternal–faetal microchimerism (40). Foetal cells enter the pregnant woman via the placenta, and while many of these cells are cleared from the body after birth, some integrate into maternal tissues and can persist for decades (41–43). Cells transferred from a foetus to a woman during pregnancy may subsequently be passed on to future foetuses in later pregnancies (44).

Maternal cells similarly enter the foetus via the placenta and become integrated throughout the body (44). These cells may persist into adulthood (45) and can be transmitted intergenerationally by female offspring (44). Such cellular migrations occur regardless of whether the foetus is conceived using the woman's own oocyte or that of another woman (46). Although more cells migrate from the foetus to the mother than *vice versa* (47), the effects of these chimeric cells may be greater in infants due to their stage of development (48). The *in utero* environment women provide also influences genetic expression in their children via the process of epigenetics (49). Through this and other mechanisms the lifelong health of children is affected by the woman who gestated and birthed them (50).

The impact of separating a newborn infant from the woman who gestated and gave birth to them remains unknown. It is often claimed that an adult intention to parent or not is of central importance in surrogacy arrangements (51); however, newborn infants are not capable of understanding adult motivations. Moreover, the behavioural research described earlier reflects that newborns have an expectation and desire to be with the woman who gestated and birthed them. Of course, infants can and do develop relationships with others who care for them. Nonetheless, at the very least, it should be recognised that separation from the mother who gestated and birthed them is a serious matter for a newborn infant. Evidence to support that gestation and birth have long-term importance can be observed in the experiences of adopted individuals. Despite equal gamete contributions from each parent, adoptees are much more likely to seek information about and search for their mother (52–55).

## 5 Language of surrogacy positions infants as “blank slates”

The concept of the “blank slate” underpins the proposition that women who gestate and give birth to infants or provide oocytes in surrogacy arrangements are not mothers. The blank slate (or *tabula rasa*) refers to the idea that humans are born without innate characteristics and are shaped entirely by their postnatal experiences (56). In the mid-twentieth century, this concept provided the foundation for practices such as closed infant adoption and anonymous donor conception. In the case of adoption, removing a newborn from an unmarried (therefore “unfit”) girl or woman at birth and giving them to a respectable married couple to raise was considered beneficial for both the child and society (57). The blank state dictated adoptive parents would determine the character and life of a child and that they were born from another woman was irrelevant (58). Adoption permanently, physically and legally, separated infants from their mother while cementing them within adoptive families “as if born to” them (59). Anonymity and secrecy were practiced with the fact of adoption usually concealed from children and knowledge of biological parentage prohibited by law (60). Similarly, secrecy was central to medically-arranged donor conception with gamete provision anonymous and parents advised not to disclose the manner of conception to their children (61). The underlying premise was again, that infants could be raised “as if” they were the biological child of both parents (62). It was assumed that neither adoptees nor donor conceived children would notice that there was anything different about their circumstances, view the fact of their origins as significant, or ever want or need to know who their biological parents were.

However, for many adoptees and donor-conceived people who perceived, were informed of, or found out about their origins, the anonymity of and separation from one or both biological parents mattered enormously and was often deeply distressing (61, 63, 64). In addition, research on identical twins separated at birth has demonstrated that, rather than being a minor factor in child development, genetics plays a substantial role in shaping personality, temperament, and interests (65). From the 1980s onwards, both adoptees and donor conceived people advocated they had rights to knowledge of their origins, to knowledge of the identity of their parents and the opportunity to contact and be in relationship with biological family (61, 66). Many jurisdictions have therefore removed secrecy from adoption, prohibited anonymous gamete donation, and promoted honesty and openness about origins from birth for both adopted and donor-conceived children (67–71). UNICEF has also affirmed that children born via surrogacy have the right to know their origins, as well as rights to identity and family relations, under the United Nations Convention on the Rights of the Child (72).

## 6 Language used by those born via surrogacy and donor conception

Very little research has considered the experiences of people born via surrogacy; however, existing evidence suggests that they may often view the woman who gestated and gave birth to them as a mother. The terms used by children reflect notions of motherhood, such as “mum,” “mummy tummy,” “surrogate mother,” “real mum,” and “birth mum” (73, 74). In the only published case study of an adult born via surrogacy, the term “birth mother” is used (75). Adults born via surrogacy who have been quoted in the media have used terms such as “surrogate mum,” “biological mother,” and “mum” (75, 76).

Research involving donor-conceived people is more extensive and indicates that gamete providers are commonly referred to using language that recognises parental status. Children have used terms such as “daddy,” “father” “kind of daddy,” “egg mum,” and “mum” (29, 73) while adults have spoken of their “biological father,” “dad,” “father,” “donor dad,” and “genetic father” (62, 64, 77). Studies of adolescents and adults conceived via donor sperm have found that between one-third and two-thirds use descriptors such as “father” or “dad” (62, 77, 78). Despite equal gamete contributions from each parent, adoptees are much more likely to seek information about and search for their mother than their father.

Caregiving parents play an influential, but not determinative, role in shaping the language children use to describe those who provided the gametes for their creation. Children often use the same terms as their caregiving parents, including those not reflective of parental status (29). However, they may also independently interpret the nature of these relationships and use descriptors like “father” and “daddy,” even when their parents attempt to discourage such terminology (29). Some parents demonstrate low tolerance for dissent, as one mother stated, “I'm careful not to use like, ‘Donor mother.’ And I will, pardon my French, shoot that shit down and I have” [(31), p. 483]. Such relationship denial, overtly or in language choice, risks inappropriately burdening children with parental insecurity and may present a barrier to identity development. Reflecting this, a study of 560 donor-conceived young adults found that more than half indicated that they had “worried that if I try to get more information about or have a relationship with my sperm donor, my mother and/or the father who raised me would feel angry or hurt” (79).

## 7 Discussion: recognising the multiplicity of mothers in surrogacy

While adults may claim that children born via surrogacy have no or only one mother, it is undeniable that they have at least one and as many as three mothers; genetic, gestational and birthing, and caregiving. The experience of adoption and donor conception indicates it is beneficial to children born via surrogacy and their caregiving parents for discussions about origins and family to begin early in life and be child-focussed and honest (80, 81). Concerns that such openness might undermine caregiving parents are unfounded; research shows that openness in adoption, both in terms of family contact and communication, is associated with better self-esteem and fewer behavioural problems in children (82). Similarly, openness with children conceived via donor conception from an early life is associated with more positive family relationships and greater adolescent well-being (81). Children who are adopted or donor-conceived do not have difficulty understanding their multiple families and the different roles that different parental figures play in their lives (83, 84).

Drawing on insights from adoption, communication that not only includes honest information-sharing but also provides space for children to express their feelings and for parents to be empathic and sensitive towards those feelings is beneficial (80). To do this, parents need to have sufficiently processed any feelings of grief or insecurity arising from infertility or from not being able to gestate and give birth to their child. Additionally, I argue, they need to respect in language the maternal nature of the relationship between the woman who gestated and birthed their child, and if relevant, provided the oocyte for their conception.

Terminology like “donor mum” or “genetic mum” can be used to describe the woman who provided the oocyte for a child's conception (31). Similarly, the woman who gestated and gave birth to the child may be referred to as the “gestational mother” or “birth mother.” When caregiving parents are comfortable discussing these other mothers, children are more likely to be able to do so as well. One mother via surrogacy spoke of “my son's other mothers” and her child thus expressed no difficulty in saying, “I have three moms: the egg donor, the one who carried me, and you, Mommy” [(27), p. 40]. Of course, it is also important for women who gestate and give birth in surrogacy arrangements or who provide oocytes to be aware that even if they do not consider themselves to be mothers, this may not be how the children perceive them. All women who contribute to the birth of a child via surrogacy should be counselled to understand that children may very well view them as mothers.

Recognising that women who provide oocytes or gestate and birth infants in surrogacy are mothers may have the effect of reducing the willingness of individuals to participate in surrogacy. However, this should not be viewed as a disadvantage; rather, it assists in informed decision-making. It may also help promote post-birth practices that are significant to children, including ongoing contact with all mothers and families. While much can be drawn from the experiences of adoption and surrogacy, there remains a need for further research into the experiences of individuals born via surrogacy.

## Data Availability

The original contributions presented in the study are included in the article/Supplementary Material, further inquiries can be directed to the corresponding author.
